# Supplementary material for: Neither exogenous, nor endogenous: Evidence for a distinct role of negative emotion during attentional control
Source: PLoS One. 2025 Oct 14;20(10):e0319888. doi: 10.1371/journal.pone.0319888 (PMC12520368; doi:10.1371/journal.pone.0319888)
Supplement: S1_File — (PDF) [file pone.0319888.s001.pdf]

– SUPPLEMENTAL MATERIALS –

ACC in Experiment 1

For the ACC (see Figure S1), The three-way repeated-measures ANOVA revealed a marginally significant interaction between Validity-V and Validity-E ($F_{1,38} = 2.907$, $p = 0.096$, $\eta_p^2 = 0.071$). None of the post hoc tests reached significance. All other effects were non-significant (all *Fs* < 3, *ps* > 0.1).

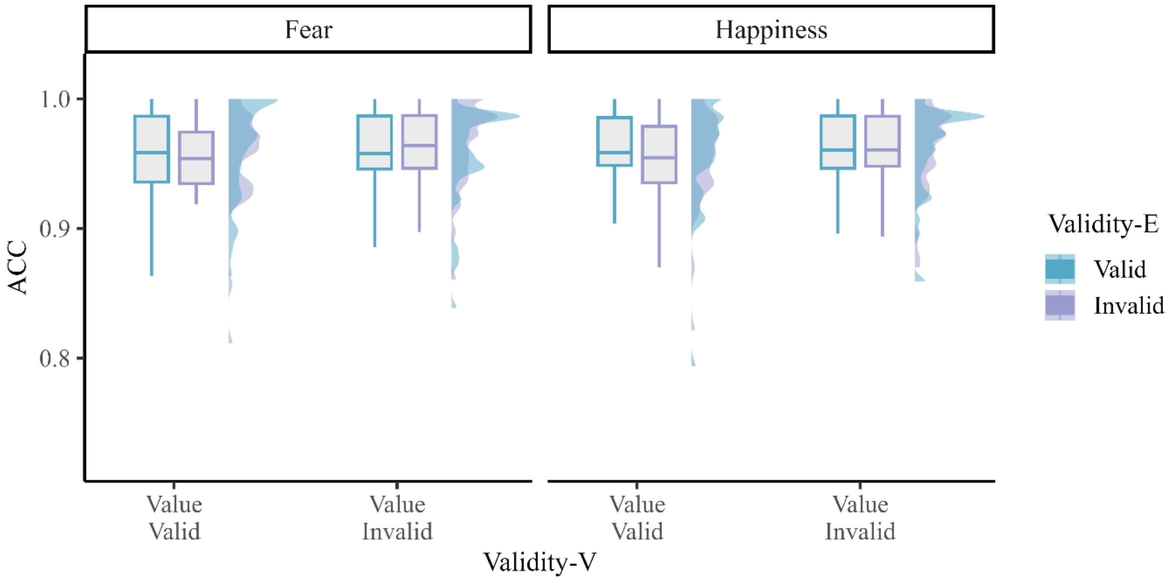

Figure S1: ACC results in Experiment 1. In each graph, the mean performance is shown in a boxplot along with their distribution (half-density with color).

ACC in Experiment 2

The three-way repeated-measures ANOVA revealed a significant main effect of Emotion ($F_{1,40} = 4.730$, $p = 0.036$, $\eta_p^2 = 0.106$; $BF_{incl} = 1.822$ suggesting anecdotal evidence for including this effect), showing a higher ACC for happiness than fear (Figure S2). The interaction between Validity and Probability was marginally significant ($F_{1,40} = 3.108$, $p = 0.086$, $\eta_p^2 = 0.072$; $BF_{incl} = 1.822$ suggesting anecdotal evidence for including this effect). None of the post hoc tests were significant. All other effects were non-significant (all *Fs* < 4, *ps* > 0.1).

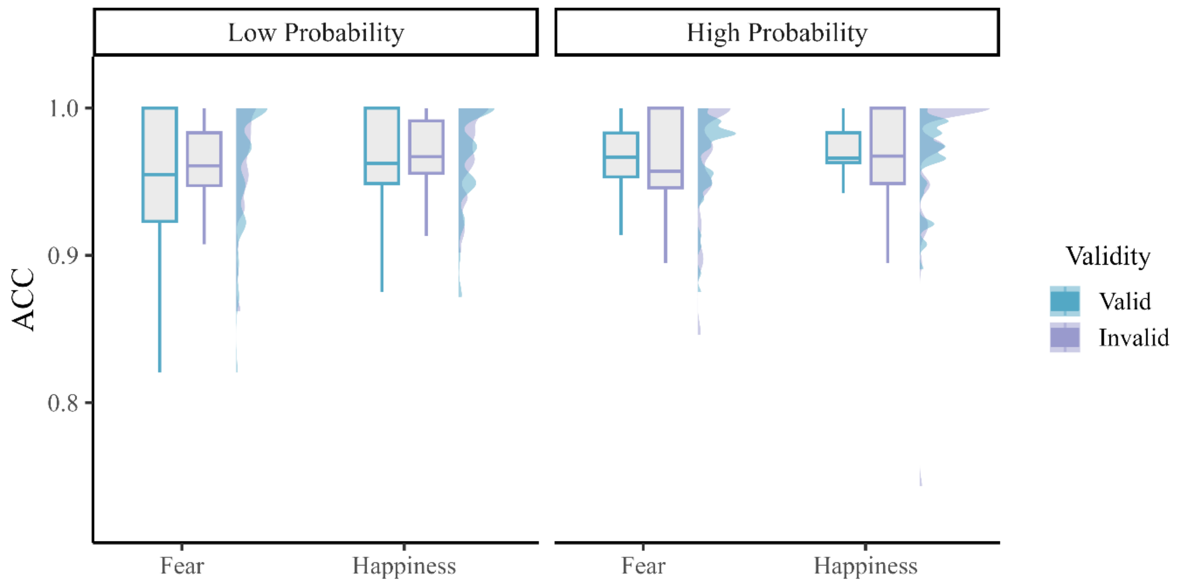

Figure S2: ACC results in Experiment 2. In each graph, the mean performance is shown in a boxplot along with their distribution (half-density with color).

Correlation between awareness and ABSes (based on ACC) in Experiment 2

For the ABSes based on ACC, in the low probability condition, awareness marginally correlated (negatively) with fear (r = -0.269, p = 0.088) but not with happiness (r = 0.144, p = 0.368). In the high probability condition, neither fear (r = -0.136, p = 0.395) nor happiness (r = -0.060, p = 0.707) was significantly related to awareness.

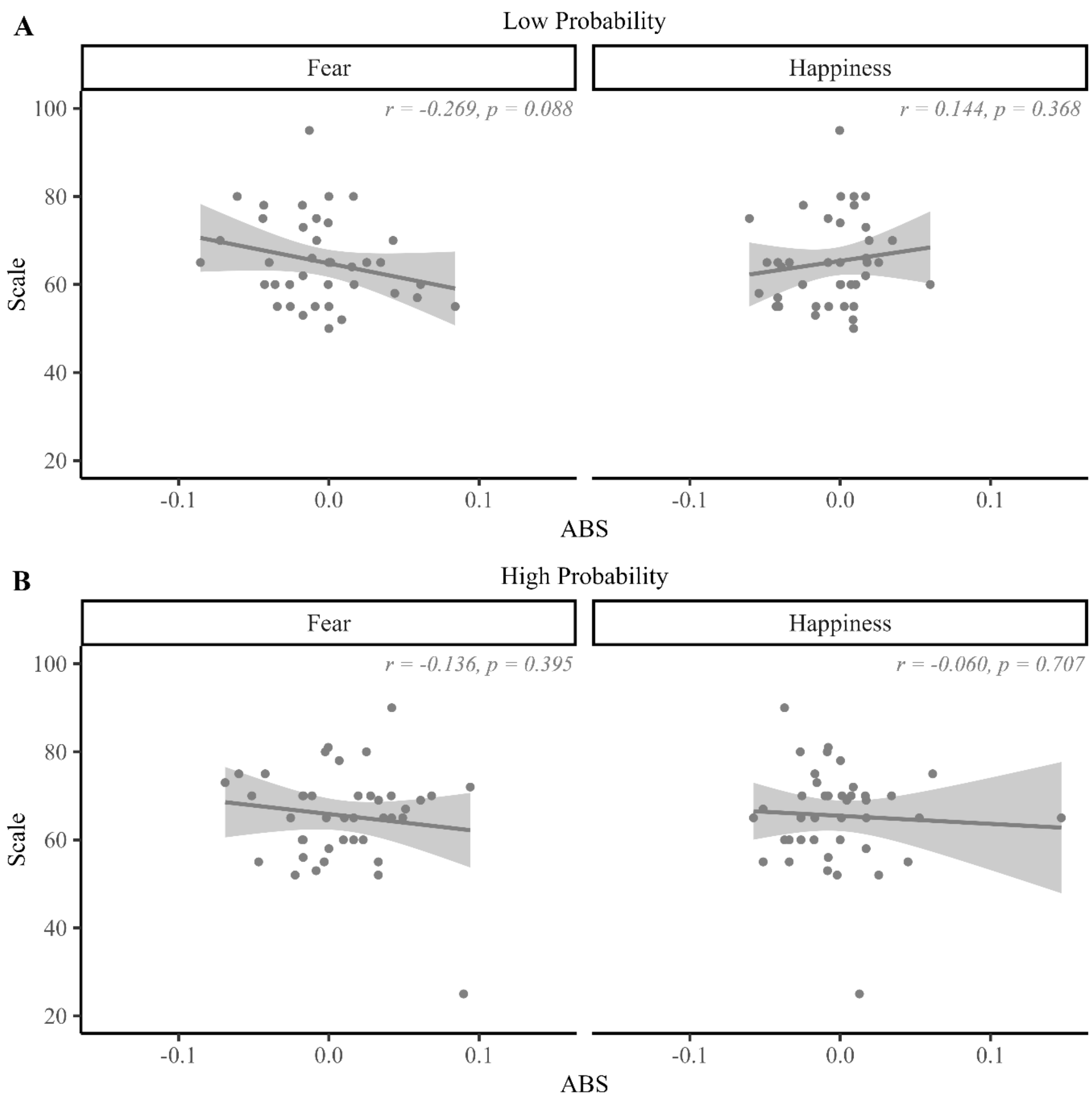

Supplementary Figure S3: Correlations between awareness and the ABS (based on ACC) separately for each emotion and probability. Each correlation is shown using a scatterplot with 95% CI.

Gender effect in Experiments 1 & 2

To assess the possible role of Gender on emotional attention, we included it as a covariate in the statistical analyses, for each experiment separately. Because our two samples were markedly unbalanced (with a majority of female participants included and tested), some caution is however needed in the interpretation of these results. For the RTs in Experiment 1, the

ANOVA showed that the interaction effect between Emotion and Validity ($F_{1,38}$ = 5.099, p = 0.032, η_p^2 = 0.121) was significant. The main effect of Gender and all interactions between Gender and the other experimental factors were non-significant (all F < 2, p > 0.1). In Experiment 2, the results showed non-significant main effects for the RTs: Emotion (F = 0.001, p = 0.992), Validity (F = 1.027, p = 0.317), Probability (F = 0.003, p = 0.984), or Gender (F = 1.078, p = 0.306). Likewise, all interactions between Gender and the other experimental factors remained non-significant (all F < 3, p > 0.1).

Results for the fixation maps

We used the iMap Toolbox to analyze (1), using a linear mixed model (LMM), eye fixations. The fixation durations were projected into the two-dimensional space according to their X and Y coordinates at the single-trial level. We smoothed the fixation duration map by convoluting it with a two-dimensional Gaussian Kernel function at 1° of visual angle. We selected the estimated method and rescaled the fixation map to 205*154 pixels (scale parameter: 0.2) to reduce computational time. Importantly, in both experiments, no significant differences between conditions were found, suggesting that eye fixations were comparable at the cue and target levels (see Figures S4-7).

Results for the fixation maps at the cue level in Experiment 1

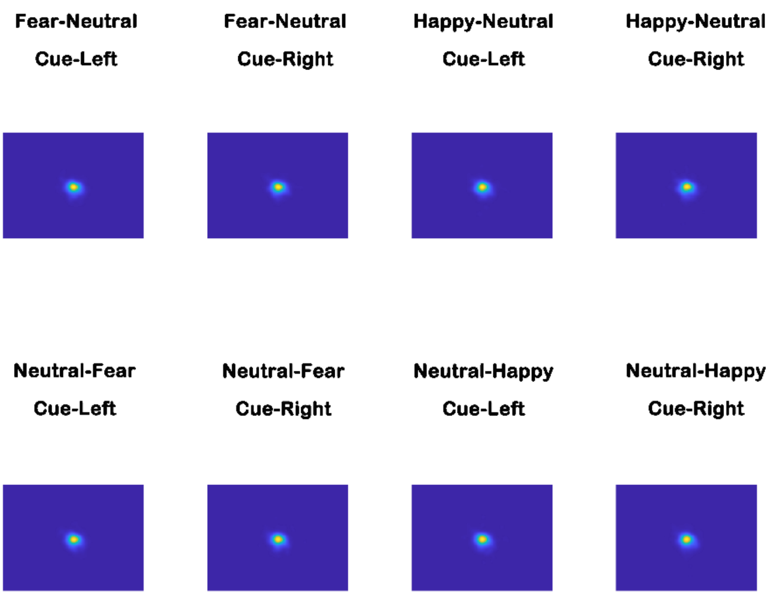

Figure S4: Results for the fixation maps, separately for each condition. The hotter the color, the more fixations at that location were made (please note that iMap4 does not provide a color scale since these fixation maps are mostly descriptive). As can be seen, participants kept fixation at the cue level and this behavior was similar for all conditions in Experiment 1 (as shown by a statistical analysis).

Results for the fixation maps at the target level in Experiment 1

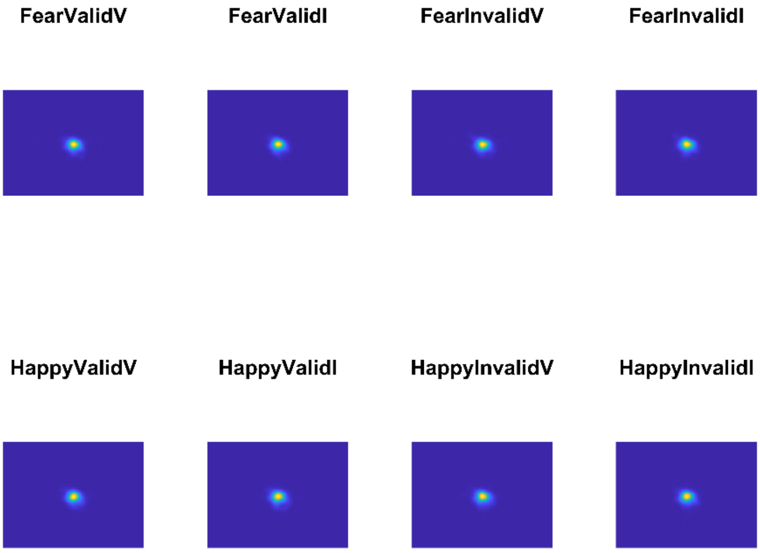

Figure S5: Results for the fixation maps, separately for each condition. The hotter the color, the more fixations at that location were made (please note that iMap4 does not provide a color scale since these fixation maps are mostly descriptive). As can be seen, participants kept fixation at the cue level and this behavior was similar for all conditions in Experiment 1 (as shown by a statistical analysis).

Results for the fixation maps at the cue level in Experiment 2

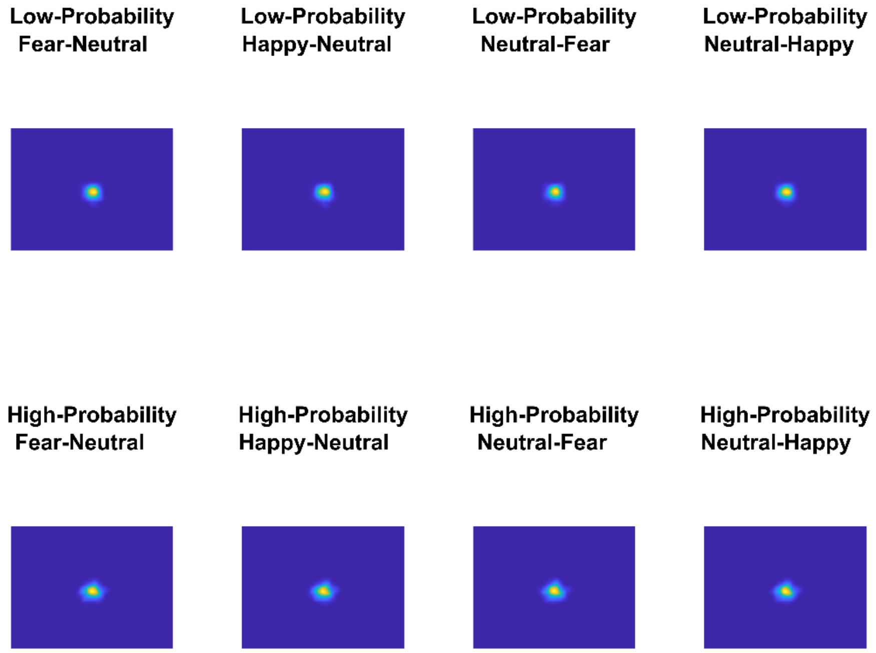

Figure S6: Results for the fixation maps, separately for each condition. The hotter the color, the more fixations at that location were made (please note that iMap4 does not provide a color scale since these fixation maps are mostly descriptive). As can be seen, participants kept fixation at the cue level and this behavior was similar for all conditions in Experiment 2 (as shown by a statistical analysis).

Results for the fixation maps at the target level in Experiment 2

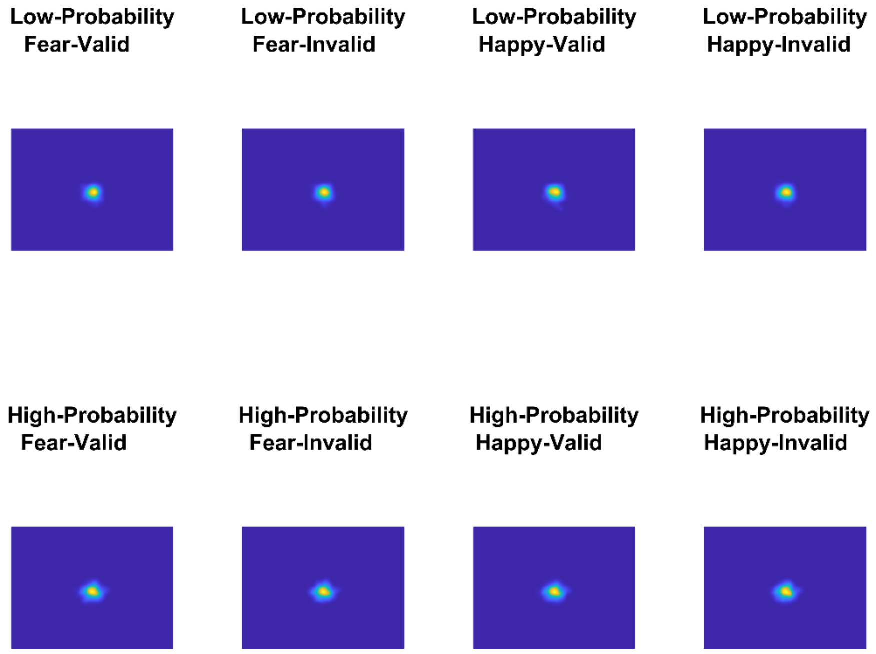

Figure S7: Results for the fixation maps, separately for each condition. The hotter the color, the more fixations at that location were made (please note that iMap4 does not provide a color scale since these fixation maps are mostly descriptive). As can be seen, participants kept fixation at the target level and this behavior was similar for all conditions in Experiment 2 (as shown by a statistical analysis).

Reference

1. Lao J, Miellet S, Pernet C, Sokhn N, Caldara R. iMap4: An open source toolbox for the statistical fixation mapping of eye movement data with linear mixed modeling. Behav Res Methods [Internet]. 2017;49(2):559–75. Available from: https://doi.org/10.3758/s13428-016-0737-x
