# Supplementary material for: Neither exogenous, nor endogenous: Evidence for a distinct role of negative emotion during attentional control
Source: PLoS One. 2025 Oct 14;20(10):e0319888. doi: 10.1371/journal.pone.0319888 (PMC12520368; doi:10.1371/journal.pone.0319888)
Supplement: Attachment — Submitted filename: Response to Reviewers.docx [file pone.0319888.s003.docx]

**Comments to the Author**
Reviewer #1: Comments to the authors:

This manuscript targets an equally important and interesting research topic. It is very well written, concise, comprehensible and transparent. It presents a sound method and offers new insights into the cross-over field of attentional and emotional processing. Therefore, I have only very view minor suggestions for improvement. Inclusion of most of those, in my opinion, do not necessarily require another review but solely a close read-through by the editor. I would suggest another review round, if new analyses and interpretation would be required, please see my comment on gender distribution. Please find attached my few comments:
**Response:** We thank the reviewer for their positive evaluation of our work and manuscript. We have revised the manuscript according to their valuable comments.

1. - Abstract: “target’s location at the block level” – without the exhaustive description of the paradigm - you nevertheless offer at a later point – it could be difficult for readers to know what “block level” means. Consider rephrasing it using e.g. condition (like yourself used later in the manuscript) or similar.

**Response:** Thank you for this suggestion. We have rephrased this sentence.

Original:
“fear, unlike happiness, could bias spatial attention in a top-down manner, yet only when participants were aware of the association created between the emotional cue and target’s location at the block level. ”

Revised:

“fear, unlike happiness, could bias spatial attention in a top‑down manner, but only when participants were aware of the association created between the emotional cue and target’s location, leading to an enhanced validity effect in the high probability condition but invalidity effect in the low probability one.”

2. - Introduction: page 4, line 61 – “(2) value is an integral part…”, consider putting “whether” before “value” for completion of the research question

**Response:** Thank you for this suggestion. However, to maintain the parallelism between these two conditions, we have revised the sentence using “when…when” rather than “whether.”

Original:
However, two unanswered questions are how is attentional control achieved when (1) salience and value directly compete with one another for selection, and (2) value is an integral part or component of the goal.

Revised:

“However, two unanswered questions are how is attentional control achieved (1) when salience and value directly compete with one another for selection, and (2) when value is an integral part or component of the goal.”

3. - Introduction: page 6, paragraph starting with line 124 – please consider explaining your previous study in more detail for better comprehension of itself and the topic of the current manuscript.

**Response:** Thanks for your suggestion. We have substantially expanded the description of our previous study to provide readers with a clearer context:

Original:

In this previous study, we found that in the absence of induction trials (i.e. baseline DPT), participants were faster for fear valid than fear invalid trials, but also symmetrically for happy invalid than happy invalid trials (even though emotion was never predictive of target’s location), suggesting that negative emotion, as opposed to fear or threat per se, actually captured attention in this task.

Revised:
“In this previous study, we compared attentional biases to emotional faces using a DPT alone where emotion was never goal-relevant (Experiment 1) or made directly task-relevant by means of induction trials (Experiments 2-3). We found out that, in the absence of induction trials (Experiment 1), negative faces captured attention, with faster target processing when it appeared on the same side as the preceding fearful face (i.e., fear-valid trials) compared to the opposite side where the neutral face was shown (i.e., fear-invalid trials), but also when it appeared on the side of the preceding neutral face (i.e., happy-invalid trials) compared to the happy face (i.e., happy-valid trials), suggesting that negative emotion, as opposed to fear or threat per se, actually captured attention in this task”

4. - Introduction: page 6, line 127 – please whether a typo occurred: “but also symmetrically for happy invalid than happy invalid trials”. Furthermore, without further description of the study itself, I do not fully understand, what “symmetrically” means in this context

**Response:** Thanks for pointing that out. See our response to point #3 raised here above. We have rewritten that paragraph. We believe this revised wording addresses both your request for more detail on our previous study as well as the typo/unclear use of the adverb “symmetrically.”

5. - Section “Participants” – Your (otherwise sound) samples present an unbalanced gender ratio (17% and 19%, respectively). I would suggest you include the (preferably absent) effects of gender on the investigated specific attentional and emotional processes in the introduction section. If gender effects cannot be ruled out, consider including it as a covariate in your analyses.

**Response:** We appreciate the reviewer’s suggestion regarding the potential influence of gender. To address this concern, we re-ran our statistical analyses including Gender as a covariate (we added these results in the supplementary material section). While some of the originally significant effects decreased and became marginally significant, the overall pattern of results remained, however. This attenuation is likely due to the inclusion of Gender as an additional factor, which is imbalanced and reduces statistical power, rather than indicating a systematic modulation of emotional attention depending on it. More importantly, the results consistently show no significant main effect of Gender and no significant interactions between Gender and the other experimental factors, indicating that Gender did not influence systematically the observed attentional effects. Given these findings, we interpret the main results with caution and directly acknowledge the unbalanced gender ratio as a potential limitation of our study in the revised manuscript on p.27.

6. - Procedure: page 11, paragraph starting with line 238 – Please consider putting the information regarding the STAI into the participants section

**Response:** As suggested, we have moved the details regarding the STAI from the Procedure section to the Participants section.

7. - Discussion: Page 19, line 418 – Please explain / elaborate the construct of IOR to the readers

**Response:** Thank you for your suggestion. We have added a brief description of IOR so that readers unfamiliar with this term and attentional effect can follow our argument.

Original:
“Because of this invalidity effect, one could argue that Inhibition of Return (IOR) could drive this Validity-V effect.”

Rivised:

“Because of this invalidity effect, one could argue that Inhibition of Return (IOR) could drive this Validity-V effect. IOR refers to the inhibition of a previously explored location to facilitate the orienting towards novel locations [1]. This is typically expressed by slower RTs for valid than invalid trials when a long SOA (i.e., above 300 ms) between cue and target is used.”

Reviewer #2: This manuscript presents a potentially valuable contribution by proposing a novel priority map to explain spatial attention biases involving both exogenous and endogenous factors. The research question is timely and thought-provoking, and several aspects of the manuscript are well-articulated. However, there are important areas where greater clarity and theoretical integration are needed to enhance the manuscript’s rigor and replicability. In particular, the comparison between Experiments 1 and 2 would benefit from a more thorough theoretical and methodological justification, especially in light of the change in target type (square vs. dot). This shift introduces potential perceptual confounds that may influence reaction times independently of attentional bias. Addressing this limitation explicitly would strengthen the interpretation of the findings and support more valid cross-experiment discussions (as is central to the authors’ proposed revision of the priority map formulated in Figure 5). Some factors are inconsistently labeled and insufficiently explained across the Results and Discussion sections, which complicates interpretation and may obscure key effects. Additionally, while the inclusion of the STAI is potentially relevant, its theoretical role in the current study remains underdeveloped and would benefit from clearer motivation and discussion. Overall, with careful attention to the issues noted—particularly regarding experimental design, factor labeling, and theoretical framing—the manuscript has the potential to make a meaningful contribution, particularly if revised for a more specialist publication outlet.

**Response:** We thank the reviewer for their positive evaluation of our manuscript, as well as the thoughtful suggestions made to improve it further. We performed a point-to-point revision of it according to them (see here below for details).

Introduction

1. p.4: “For example, fear (conveyed by a fearful face) can enhance contrast sensitivity (24) and this perceptual effect appears to be mostly mediated by the processing of coarse/low-spatial frequency information (25).” Similar claims have also been made with respect to the facilitative effects of processing happy facial expressions:
Calvo, M. G. and Marrero, H. (2008). Visual search of emotional faces: The role of aﬀective content and featural distinctiveness. Cognition and Emotion 22, 1-25.
Calvo, M. G. and Nummenmaa, L. (2008). Detection of Emotional Faces: Salient physical features guide eﬀective visual search. Journal of Experimental Psychology: General 137, 471-494.
The authors are encouraged to review this work and incorporate it alongside the arguments presented on page 4 in order to add a more nuanced perspective on the topic.

**Response:** Thanks for these thoughtful suggestions. We have revised the manuscript to convey a more nuanced discussion of this important topic and now directly refer to these earlier studies suggested by the reviewer. The revised text reads as follows:

“For example, fear (conveyed by a fearful face) can enhance contrast sensitivity (24) and this perceptual effect appears to be mostly mediated by the processing of coarse/low-spatial frequency information (25). However, happy faces, for which the upward-curved mouth provides an important diagnostic (low-level) feature, can also facilitate visual processing and bias spatial attention compared to neutral faces (26,27). Hence, although threat-related stimuli can be prioritized during attentional selection, probably due to their enhanced evolutionary relevance (28–30), positive stimuli can also bias spatial attention under specific circumstances.”

2. p.4: It would be helpful for the authors to specify here the tasks used to assess faster/better processing of threat-related stimuli in the following statement: “as revealed by faster/better processing for them compared to neutral or even positive stimuli (26–28).”

**Response:** Thanks for this suggestion. In the revised manuscript, we now specify the experimental tasks that have demonstrated faster threat detection.

Original:

These results align with many other ones showing that threat-related stimuli capture attention involuntarily, as revealed by faster/better processing for them compared to neutral or even positive stimuli [2–4].

Revised:

These results align with many other ones showing that threat-related stimuli capture attention involuntarily, as revealed by faster/better processing for them compared to neutral or even positive stimuli in various tasks and contexts [2–4], including visual search [3,5], attentional blink [6,7], cueing [8,9], or dot-probe task (DPT) [10,11].

3. p.5: DPT abbreviation should follow “dot-probe task”.

**Response:** As suggested, we have added it.

4. p.5: The authors should clarify whether low-level physical change was manipulated for happy faces as well in the following statement: “As a control condition, we used happy
faces.”

**Response:** We clarified this issue and confirmed it was indeed the case. In Experiment 1, the low-level change in terms of luminance was exactly the same for fearful and happy faces. We have added the following sentence to make this explicit:
“As a control condition, we used happy faces. In this condition, the same abrupt luminance change occurred on one side of the display, as was done with fearful faces, eventually yielding a comparable exogenous cueing for these two emotion categories.”

5. p.5: The authors need to describe in more detail how induction trials were used to activate a specific goal in their description of the following: “For example, Vogt et al. (44) used a DPT in combination with induction trials meant to activate a specific goal and found that negative stimuli only captured attention when they were directly task-relevant and hence goal-relevant (40,44,48– 50).”

**Response:** Thank you for this suggestion. In the revised manuscript (page 5), we have clarified this point:
“For example, Vogt et al. (44) used a DPT in combination with induction trials meant to activate a specific goal and found that negative stimuli only captured attention when they were directly task-relevant and hence goal-relevant (40,44,48–50). More specifically, during the induction trials, the participants were asked to press a key whenever a specific and pre-defined (emotional) stimulus was presented but to withhold responding to other stimuli, making this former stimulus task and hence goal relevant.”

6. p.6: The authors should clarify how the low vs. high probability conditions are defined, as the current explanation is confusing. The sentence suggests that in both conditions the target appears on the same side as a specific face type (neutral or emotional) in 75% of trials, which makes it unclear what distinguishes the two conditions. Please specify more clearly how the probability differs between conditions and which face type serves as the probabilistic cue in each case. “In agreement with these instructions, in the low probability condition, the target appeared on 75% of the trials on the same side as the neutral face while in the high probability condition, it appeared on 75% of the trials on the same side as the emotional face instead.”

**Response:** Thank you for this suggestion. We have revised the manuscript on page 6 to clarify this issue. Low vs. High probability simply refers to the likelihood (either low or high) that the target appeared on the same side as the emotional face at the cue level. Accordingly, in the low probability condition, the target appeared on 75% of the trials on the same side as the neutral face, while it was shown on 25% of the trials on the same side as emotional face instead. In comparison, in the high probability condition, this mapping was reversed: it appeared on 75% of the trials on the same side as the emotional face, whereas on 25% of the trials, it was shown on the same side as the neutral face.

Methods
7. p.7: What is ACC? It is the first time this abbreviation is used.

**Response:** We apologize for this. ACC simply means Accuracy and we have abbreviated it upon its first occurrence in the text on p.8.

8. p.8: The authors should specify the Ekman and Friesen dataset here instead of “Ekman dataset.”

**Response:** Thanks for pointing this out. We revised the wording accordingly.

9. p.8: Possible typo: the tick one/the thick one.

**Response:** Thank you for noticing this typo that we have corrected.

10. p.9: The authors need to explain why were different SOAs used. The analysis by SOA should be included in a dedicated sub-section of the Results, rather than the supplemental section in order to facilitate the interpretation of the results on pages 19-20 in the Discussion.

**Response:** We thank the reviewer for this comment and suggestion. As we now better explain on p. 11 in the revised manuscript, we mostly used these different/variable SOAs to prevent temporal attention effects and in keeping with Pourtois et al. (2004) where this variability was introduced (with the DPT). As a result of it, the participants could not easily anticipate the actual onset of the target.

Moreover, as suggested by the reviewer, we moved the SOA control analysis from the supplementary materials section into the Results section (of the main text).

11. p.9: The level of detail regarding the instructions provided to participants is insufficient: “Participants were asked to discriminate the shape of the target, either a diamond or a square, as accurately and quickly as possible. Speed was emphasized and it provided the main dependent variable in both experiments.” Please provide full details regarding what the participants were required to do when they saw a face on each trial, how was speed emphasised to participants and whether any feedback provided.

**Response:** Following the reviewer’s suggestion, we have provided additional details about the instructions given to the participants. Regarding the DPT used in our study, we used a simple visual discrimination task based on the shape of the target (i.e., to discriminate whether the shape of the target was either a diamond or a square). Importantly, participants were not required to engage with or process (emotional) stimuli at the cue level, as typically done with the DPT in the existing literature. To emphasize speed, participants were explicitly instructed about this feature before the start of each block, with the use of a specific instruction display: “Respond as quickly and accurately as possible!”. Additionally, a strict 2-second response window was enforced for each trial. If participants failed to respond within this window, the trial was automatically terminated, and the task proceeded to the next trial. No trial-by-trial feedback was provided.

In the revised manuscript on p.11 these elements related to the instructions are now conveyed explicitly:

“Each trial began with a fixation cross shown for 500 ms, followed by the emotional cue (i.e., a pair of faces) shown for 100 ms either within the cued frame (i.e. thick rectangle) or within the uncued one (i.e., the thin rectangle), with an equal probability on either the left or right side. Participants were not required to process the stimuli at the cue level. After a short, variable, and equiprobable interval (100, 150, 200, 250, or 300 ms), the target was presented for 150 ms. Participants were asked to discriminate the shape of the target, either a diamond or a square, as quickly and accurately as possible. Speed was emphasized via on‑screen instructions shown before each block and it provided the main dependent variable of interest in both experiments. A maximum response window of 2s was imposed on every trial; if participants failed to respond within 2s, it was automatically terminated, and the task proceeded to the next trial. After the response (or 2000 ms in case of no response), a 500ms interval was used, before the next trial started. ”

12. p.10: When Experiment 2 is introduced on page 10, the authors should clarify whether luminance was also manipulated.

**Response:** As suggested by the reviewer, we clarified that in Experiment 2 (and unlike Experiment 1), luminance (and hence exogenous cueing) was not manipulated.

13. p.10: “a visual analog scale (VAS). This VAS consisted of a continuous scale ranging from 0 (no awareness of the association) to 100 (full awareness of the association). Hence, higher scores on this VAS indicated greater awareness at the subjective level. At the beginning of each session, the participants received specific instructions (on screen).” It is not clear what type of awareness the VAS measures. If participants received instructions about the probability of a target appearing after an emotional or neutral face at the beginning of each block, what exactly were the participants asked to reflect on in the VAS?

**Response:** Thank you for pointing out this ambiguity, which we have clarified on p.12 in the revised manuscript.
“Each session included one practice block of 20 trials, followed by 4 experimental blocks of 80 trials. After these 4 blocks, participants’ awareness of the association between the cue and the target was probed using a visual analog scale (VAS). Using this scale, they were asked to indicate their explicit knowledge of the association created between the cue and the target. Hence, in the low probability condition, they were asked to indicate on a scale ranging from 0 (no knowledge) to 100 (full knowledge) how aware they were of the fact that the target most of the time appeared on the same side as the neutral face. Similarly, in the high probability condition, they were asked to rate their awareness of the contingency created between target location and side of the emotional face.”

14. p.10: The authors should clarify if the SOAs used in Experiment 2 were equiprobable (as described in Experiment 1).

**Response:** As suggested by the reviewer, we confirm (see revised manuscript on p.12) that the variable (and equiprobable) SOAs used in Experiment 2 were actually the same as those used in Experiment 1.

15. p.10: “the target (either a dot or a square) was shown for 150 ms at the location previously occupied by the emotional face (valid trial) or the neutral face (invalid trial).” Why was the target changed from diamond to dot in Experiment 2?

**Response:** Thank you for the careful reading of our manuscript and for noticing this inconsistency. There, we inadvertently used the word “dot”, but this was a mistake. As was done in Experiment 1, we confirm that the target consisted either of a square or a diamond. We have corrected this error in the revised manuscript.

16. p.10: Inconsistency with target description here: “diamond and their right index finger for the square”, when in the previous sentence the target is described as a dot.

**Response:** As noted here above in our response to the previous point raised by the reviewer, this was a mistake that we have corrected.

17. p.11: “At the end of the experiment, participants were asked to complete the trait version of the State-Trait Anxiety Inventory (STAI-T; see Spielberger (55)). We used the twenty-item version (Form Y-2) that assessed trait anxiety. All items were rated on a 4-point scale (e.g., from “Almost Never” to “Almost Always”). Scores range from 20 to 80, with higher scores reflecting higher levels of anxiety. The average score of the STAI was 43.80 (range 26-68; SD: 10.265) in Experiment 1 and 38.27 (range 29-63; SD: 8.155) in Experiment 2. The mean score in Experiment 2 was significantly lower than in Experiment 1 ( 78 = -2.673, p = 0.009, Cohen’s d = -0.598). Despite this significant difference, both scores were lower than the commonly used cutoff of 45 for clinical anxiety, indicating that neither group reached clinical anxiety.” What is the theoretical motivation for administering the STAI? Why is a cut-off of 45 used (and where is the citation to support it?). The authors are encouraged to consult the STAI manual for relevant associated norms and conduct relevant comparison tests with these values.

Spielberger, C. D., Gorsuch, R. L., Lushene, R., Vagg, P., & Jacobs, G. (1983). Manual for the state-trait anxiety inventory. Consulting Psychologists Press.

**Response**: On p.8 in the revised manuscript, we clarified that we administered the STAI to our participants because anxiety, as measured using this specific scale, has been shown to influence emotional attention in some previous studies [12,13]. Moreover, we also used it to assess if anxiety levels of our participants were eventually comparable between the two experiments performed, and found that they were actually (and unexpectedly) lower in Experiment 2 than 1. However, when consulting the influential work of Elaine Fox and colleagues [2,14], we found out that they could be considered low, sub-clinical anxiety. Hence, it was not our intention to use norms or specific cut-offs, but we simply wanted to compare our results for the STAI with some previous studies on emotional attention, anxiety and threat processing available in the literature.

Data Analysis

18. p. 11-12: It’s not clear what the Total and Correct trials column refers to in Tables 1 and 2.

**Response:** Thank you for pointing that out. We improved the legends of Tables 1-2 to explain this better.
“Note: Standard deviation is shown in parentheses. “Total” refers to the total number of trials presented per condition; “Eye‐tracking correction” to the mean number of trials kept after exclusion of excessive eye‐movements; “Correct trials” to mean number of trials kept with correct responses.”

19. p.13: The way the factors are labelled is a little confusing: In the Data Analysis section on page 12 one of the factors is presented as: “Validity-V (here “V” refers to value of the face: the target replaced either the emotional or neutral face at the cue level)”, but in the Results section of Experiment 1 on page 13 it is described as “ anecdotal evidence for including this effect), showing faster RTs for Invalid than Valid trials (see Fig 2B).”

**Response:** Thank you for pointing out this apparent confusion. To resolve it, we have better explained on p.14 what the factor “Validity‑V” actually refers to:

Original:

“Validity-V (here “V” refers to value of the face: the target replaced either the emotional or neutral face at the cue level), ”

Revised:
“Validity-V (here “V” refers to value of the face: the target replaced either the emotional or neutral face at the cue level, corresponding to valid and invalid trials, respectively).”

20. p.13: Could the following be contextualised a little better: “with faster RTs for happy than fearful trials.” Does this perhaps refer to faster response times to the target when it is preceded by happy face trials than fearful trials”?

**Response:** As correctly surmised by the reviewer, this effect indeed translated into faster RTs for trials where a happy face was used at the cue level compared to a fearful face. We revised the manuscript on p.15 accordingly.
Discussion

21. p.17: What does F_ABS refer to?
**Response:** Sorry about this omission. We clarified that “F_ABS” simply refers to attentional bias score for fear. In the revised manuscript, we have abbreviated it upon its first occurrence (on p.19).

22. p.18: The labelling of factors is confusing and difficult to follow throughout the discussion. The authors are encouraged to come up with a more meaningful way to label the factors than Validity E.

Response: Thank you for this suggestion. For consistency, we decided to retain the labels “Validity‑E” and “Validity‑V,” but we have now clarified their meaning on the p.14 to avoid confusion. Specifically, we explicitly mention that:

“Validity-E (here “E” refers to exogenous cue: the target replaced either the thick or thin rectangle at the cue level, corresponding to valid and invalid trials, respectively) ”

23. p.19: The authors should clarify whether the luminance change affected the processing of the target equally for happy and fearful faces in the following statement: “luminance change occurring at the cue level was processed as a salient event by the participant, thereby facilitating target processing when it was shown at the same location as this change

**Response**: As requested by the reviewer, we clarified this statement (see our changes on p.22). In Experiment 1, the abrupt luminance change at the cue level facilitated target processing at this location equally strongly for fearful and happy faces. This was reflected in a significant main effect of Validity-E and no significant interaction between Validity-E and Emotion.

24. p.19: The authors claim that “neutral faces are perceived as negative stimuli in this task,” How have the authors established whether this is the case? Were the neutral faces rated negatively by participants? Where is the data to support this assertion?

**Response**: Thank you for raising this important point. Even though we did not ask the participants to rate the different faces used in our experiments (mostly due to time constraints), based on independent results obtained using a similar DPT and the same emotional faces (see Xue & Pourtois, 2025a, 2025b [15,16]), we have good reasons to assume that these neutral faces were indeed perceived as somewhat negative stimuli. In these two recent studies, we used induction trials and asked participants to process explicitly the emotional content of the cue with them. The results of these induction trials clearly showed that it turned out to be more difficult for them to discriminate fearful from neutral faces (fear trials) than happy from neutral faces (happy trials). To account for this emotional effect and asymmetry (that was highly significant), we argued that these neutral faces competed with fearful faces because they were both perceived as negative somehow; a competition that was obviously decreased when these same neutral faces were shown concurrently with happy faces (and a clear contrast between negative and positive emotion likely occurred). This interpretation is not odd, but compatible with other and independent evidence available in the literature [17,18]. Hence, neutral faces, especially when presented briefly and in the periphery (as done in our experiments), could be perceived as negative stimuli. Accordingly, we assume that the same negativity bias for neutral faces occurred here, eventually explaining why the participants of Experiment 1 oriented preferentially to the side of the neutral face when it competed with a happy face. We added these important elements of clarification in the revised Discussion on p.23.

References

25. p.28: Author order correction: Ekman, P, Friesen, W.V.

**Response:** Thank you for pointing this out. We have corrected it.

Reference

1.	Klein RM. Inhibition of return. Trends Cogn Sci. 2000;4: 138–147. doi:10.1016/S1364-6613(00)01452-2

2.	Fox E. Processing emotional facial expressions: The role of anxiety and awareness. Cogn Affect Behav Neurosci. 2002;2: 52–63. doi:10.3758/cabn.2.1.52

3.	Eastwood JD, Smilek D, Merikle PM. Differential attentional guidance by unattended faces expressing positive and negative emotion. Percept Psychophys. 2001;63: 1004–1013. doi:10.3758/BF03194519

4.	Pourtois G, Grandjean D, Sander D, Vuilleumier P. Electrophysiological correlates of rapid spatial orienting towards fearful faces. Cerebral Cortex. 2004;14: 619–633. doi:10.1093/cercor/bhh023

5.	Fox E, Lester V, Russo R, Bowles RJ, Pichler A, Dutton K. Facial Expressions of Emotion: Are Angry Faces Detected More Efficiently? Cogn Emot. 2000;14: 61–92. doi:10.1080/026999300378996

6.	Anderson AK, Phelps EA. Lesions of the human amygdala impair enhanced perception of emotionally salient events. Nature. 2001;411: 305–309. doi:10.1038/35077083

7.	Schwabe L, Merz CJ, Walter B, Vaitl D, Wolf OT, Stark R. Emotional modulation of the attentional blink: the neural structures involved in capturing and holding attention. Neuropsychologia. 2011;49: 416–425. doi:10.1016/j.neuropsychologia.2010.12.037

8.	Fox E, Russo R, Bowles R, Dutton K. Do threatening stimuli draw or hold visual attention in subclinical anxiety? J Exp Psychol Gen. 2001;130: 681–700. doi:10.1037/0096-3445.130.4.681

9.	Phelps EA, Ling S, Carrasco M. Emotion Facilitates Perception and Potentiates the Perceptual Benefits of Attention. Psychol Sci. 2006;17: 292–299. doi:10.1111/j.1467-9280.2006.01701.x

10.	Lipp O V., Derakshan N. Attentional bias to pictures of fear-relevant animals in a dot probe task. Emotion. 2005;5: 365–369. doi:10.1037/1528-3542.5.3.365

11.	Mogg K, Bradley BP. Orienting of attention to threatening facial expressions presented under conditions of restricted awareness. Cogn Emot. 1999;13: 713–740. doi:10.1080/026999399379050

12.	Bishop SJ. Neurocognitive mechanisms of anxiety: an integrative account. Trends Cogn Sci. 2007;11: 307–316. doi:10.1016/j.tics.2007.05.008

13.	Eysenck MW, Derakshan N, Santos R, Calvo MG. Anxiety and cognitive performance: Attentional control theory. Emotion. 2007;7: 336–353. doi:10.1037/1528-3542.7.2.336

14.	Fox E, Russo R, Dutton K. Attentional Bias for Threat: Evidence for Delayed Disengagement from Emotional Faces. Cogn Emot. 2002;16: 355–379. doi:10.1080/02699930143000527

15.	Xue X, Pourtois G. Modulatory effects of goal relevance on emotional attention reveal that fear has a distinct value. Cogn Emot. 2024;39: 246–260. doi:10.1080/02699931.2024.2405014

16.	Xue X, Pourtois G. Neurophysiological evidence for emotional attention modulation depending on goal relevance. Sci Rep. 2025;15. doi:10.1038/s41598-025-96537-y

17.	Calvo MG, Nummenmaa L. Detection of Emotional Faces: Salient Physical Features Guide Effective Visual Search. J Exp Psychol Gen. 2008;137: 471–494. doi:10.1037/a0012771

18.	Lee E, Kang JI, Park IH, Kim JJ, An SK. Is a neutral face really evaluated as being emotionally neutral? Psychiatry Res. 2008;157: 77–85. doi:10.1016/j.psychres.2007.02.005
